# Hardening the Constitution

**Published:** 1874-03

**Authors:** 


### HARDENING THE CONSTITUTION.

Men talk about "*hardening the constitution*," and with that view, expose themselves to summer's sun and winter's wind, to strains and over efforts, and many unnecessary hardships. To the same end, ill informed mothers souse their little infants in cold water day by day; their skin and flesh, and bodies, as steadily growing rougher and thinner, and weaker, until slow fever, or water on the brain, or consumption of the bowels, carries them to the grave; and then they administer to themselves the semi-comfort and rather questionable consolation, of its being a *mysterious dispensation* of Providence, when in fact, Providence had nothing to do with it: He works no miracle to counteract our follies.

The best way I know of *hardening the constitution*, is to take good care of it, for it is no more improved by harsh treatment, than a fine garment or new hat is made better by being banged about.

